# Adherence to and persistence with zoledronic acid treatment for osteoporosis—reasons for early discontinuation

**DOI:** 10.1007/s11657-020-00733-4

**Published:** 2020-04-17

**Authors:** Anna Spångeus, Simon Johansson, Mischa Woisetschläger

**Affiliations:** 1 Department of Acute Internal Medicine and Geriatrics, Linköping University Hospital, Linköping, Sweden; 2 Division of Diagnostics and Specialist Medicine, Department of Health, Medicine and Caring Sciences, Linköping University, Linköping, Sweden; 3 Department of Radiology, Linköping University Hospital, Linköping, Sweden

**Keywords:** Osteoporosis, Bisphosphonate, Zoledronic acid, Adherence, Discontinuation, Adverse event

## Abstract

***Summary*:**

This retrospective study reports 81% long-term (> 3 years) adherence to and 77% persistence with zoledronic acid (ZA) treatment in osteoporosis patients, with ZA being costfree for patients. Eight percent of patients discontinued treatment because of adverse events (AEs), with a tendency of higher discontinuation rate in older patients.

**Purpose:**

This study investigated (1) long-term adherence to and persistence with ZA treatment in a real-world setting, (2) extent to which an adverse reaction to ZA impacted on adherence and persistence, and (3) whether there were sex or age differences in patients that had early treatment termination (ETT) due to AEs and those who adhered to the regimen.

**Methods:**

All patients treated with ZA at the Endocrinology Department at Linköping University Hospital, Linköping, Sweden between 2012 and 2017 were included. ETT was defined as < 3 ZA infusions, which was confirmed from patients’ medical records.

**Results:**

A total of 414 patients were treated with ZA, with 81% receiving > 3 ZA infusions. Three-year persistence was 77% for a treatment window of 365 days ± 90 days (75% with 365 days ± 60 days window). The most common reason for ETT was AEs (8%), followed by medical conditions (5%), biological aging (3%), and other (e.g., lost to follow-up [3%]). Most patients who discontinued treatment because of AEs reported symptoms of acute-phase reaction, and tended to be older than those who adhered to treatment (74 ± 9 vs 70 ± 13 years, *p* = 0.064). There was no difference in sex ratio between the 2 groups (85% vs 90% females, *p* = 0.367).

**Conclusion:**

Rates of long-term adherence to and persistence with ZA treatment were high with a pre-scheduled 3-year treatment regimen in the tax-financed Swedish healthcare system. AEs—mainly acute-phase reaction—were the most common reason for ETT, occurring in nearly 1 out of 10 patients.

## Introduction

Fragility fractures secondary to osteoporosis are highly prevalent, with about 9 million cases yearly worldwide [1]. Despite being associated with substantial morbidity and mortality and representing a significant economic burden for society [2–5], osteoporosis remains highly underdiagnosed and undertreated [6]. Additionally, previous studies have reported poor persistence with osteoporosis treatment [7–12].

Various cost-effective treatment options are available for osteoporosis including antiresorptive and anabolic treatments [13, 14]. Antiresorptive bisphosphonate, either oral or parenteral, is a first-line treatment in many guidelines including the Swedish [15]. Several studies have reported low persistence to and adherence with both oral and parenteral antiosteoporotic therapies, but most studies had short observation times (i.e., 12–24 months) [7–12, 14] whereas guidelines recommend a longer treatment (minimum of 3 years) consisting of yearly administration of parenteral bisphosphonate zoledronic acid (ZA) [14].

A common adverse event (AE) of ZA is acute phase reaction (APR), which occurs in about 40% of patients [16] and causes influenza-like symptoms such as fatigue, arthralgia, nausea, headache, and fever [14]. This reaction is linked to the pharmacodynamics of ZA and typically begins within 24 h after administration, and usually resolves a few days post-infusion [14, 16]. Symptoms are often mild and can be alleviated with nonsteroidal anti-inflammatory drugs (NSAIDs) or paracetamol [14], but in some patients symptoms are prolonged and less tolerable. The extent to which these symptoms interfere with patients’ willingness to continue ZA treatment is unclear.

Given the importance of compliance in maximizing the effects of therapeutic interventions, the present study investigated (1) long-term (3 years) adherence to and persistence with ZA treatment in a real-world setting, (2) the extent to which these are impacted by APR, and (3) whether patients with early treatment termination (ETT) because of adverse events (AEs) differ from those who adhered to ZA treatment with respect to sex ratio and age.

## Material and methods

### Study context

The study was carried out at the osteoporosis unit at Linköping University Hospital, Sweden. Patients were either receiving ZA treatment for severe osteoporosis or were being followed at the unit, or patients with a milder form of the disease who were being routinely followed at a primary healthcare unit but were referred to the osteoporosis unit for assistance with ZA infusion—most primary health care units in the catchment area did not handle infusions during the study period. Other specialized clinics (e.g., rheumatology clinic) administered infusions. Patients receiving bisphosphonates for cancer were not treated at the osteoporosis unit.

The healthcare system in Sweden is tax-financed and patients do not pay for ZA treatment. Three yearly infusions are routinely scheduled unless otherwise indicated. Nurses contact the patients before each treatment for blood tests and schedule an appointment for the infusion. Patients received a standardized information letter prior to ZA infusion, including recommendations of being well hydrated and using paracetamol peri-infusion (starting 1 day before and ending 2 days after).

### Study design

To identify all patients treated with ZA at the osteoporosis unit, we used the local database connected to the Cosmic intelligence software case record system (Cambio, Linköping, Sweden). The search included all patients who visited a clinic from 2012 to 2017 and who met the following three criteria: (1) coded with procedure code DT016 (“intravenous drug administration”), (2) assigned to the osteoporosis unit of the endocrinology clinic, and (3) coded for ZA in the planning system. Exclusion criteria were patients receiving ZA for reasons other than osteoporosis (e.g., Paget disease) and patients that had received < 2 ZA treatments and deceased the same or following year after the last ZA infusion (*n* = 27).

The output file included dates of ZA infusions as well as year of birth and sex. Treatments were confirmed through medical records, and patients were categorized as either ETT or fully treated (non-ETT; i.e., > 3 ZA infusions). Some patients were wrongly categorized with too few infusions in the output file, which was corrected by referring to case records. Major reasons for this were that 1 or more infusions had been performed by other clinics, or the procedure code was missing in the documentation for the treatment visit. Patients who received their first or second ZA infusion in 2017 were followed until the third ZA infusion or ETT. Adherence was defined as > 3 ZA treatments. Persistence was assigned a permissive time of 90 days in a planned 1-year interval. Fewer patients were included in the persistence analysis (*n* = 390) than in the adherence analysis (*n* = 414) due to missing dates of ZA infusion (when performed in other clinics or before 2012).

### ETT

Reasons for ETT were categorized as follows: (1) AEs (ETT-Adverse) such as acute phase reaction, but also other symptoms debuting in adjunction to the treatment and making patients unwilling to take additional ZA; (2) medical reasons (ETT-Medical) such as reduced glomerular filtration rate (GFR), termination of cortisone treatment, and lack of response to treatment; (3) biological aging (ETT-BiologicalAge)—i.e., the patient refused further treatment because of advancing age or terminal illness; and (4) other reasons (ETT-Other), e.g., failure of the healthcare unit to follow up with the patient. AEs were further subclassified into APR (ETT-APR) or other AE.

### Statistical analysis

IBM SPSS v25.0 for Windows software (IBM Inc., Armonk, NY, USA) was used for statistical analyses. ANOVA and the *χ*^2^ test were used to compare continuous and categorical variables, respectively, between groups. Kaplan-Meyer curves were used for persistence analysis, and intergroup comparisons were performed with the log-rank (Mantel-Cox) test. All statistical tests were performed at the 5% significance level.

### Ethics

The Regional Research Ethics Committee of the Faculty of Health Sciences, Linköping University approved this study (2017/507–31).

## Results

### Patients

A total of 414 patients initiated ZA infusion for osteoporosis between 2012 and 2017. The number of females who received ZA treatment was 6 times higher than the number of males (86% vs 14%); mean age at first ZA infusion was 71 ± 13 years (Table 1).

**Table 1:** Patient characteristics and comparative data for patients receiving full ZA treatment vs those discontinuing treatment because of adverse events

		Full treatment vs early treatment termination because of adverse event		
		Full treatment	ETT-Adverse^†^	*p* value
Number of patients	414	338	33	
Age at first ZA infusion, years*	71 ± 13	70 ± 13	75 ± 9	0.064
Age > 80 years at first ZA, years	26%	23%	30%	0.388
Sex (% female)	86%	85%	90%	0.367

*Age is shown as mean ± standard deviation

^†^Early treatment discontinuation because of adverse events

### Adherence and ETT

**Fig. 1:**
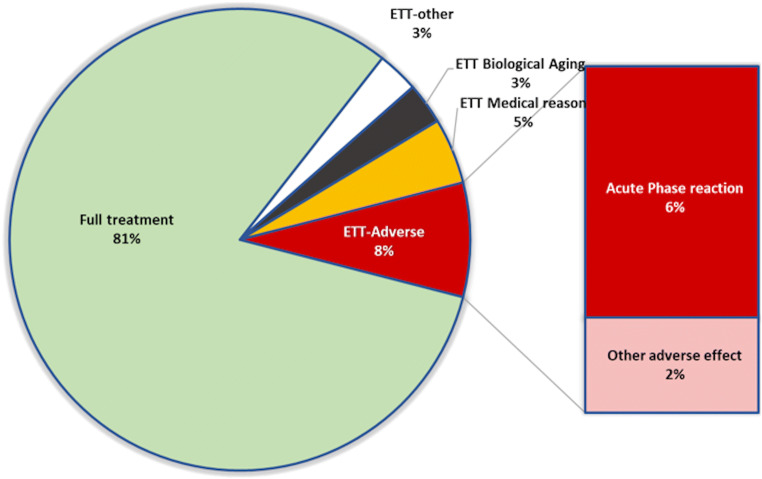
Patient treatment profile. Of the patients receiving ZA infusions between 2012 and 2017, 81% received full treatment (> 3 infusions); ETT was recorded in 19% of patients, with reasons including AEs (ETT-Adverse), medical reasons, biological aging, and other. ETT early treatment termination, AE adverse event

In total, 81% of patients received > 3 ZA infusions; the remaining 19% had ETT (Fig. 1), with AEs being the most common reason (8% of all treated patients), followed by ETT-Medical (e.g., progressive kidney failure; 5%).

Most patients in the ETT-Adverse group reported symptoms of APR including fever, muscle pain, weakness, and low energy. Other patients reported heart symptoms (atrial fibrillation and pericardial effusion) with association with ZA treatment initiation, which made patients unwilling to continue treatment. Most ETT-Adverse patients (82%) terminated ZA treatment after the first infusion and the remaining 18% after the second infusion. There was no difference in sex ratio between the non-ETT and ETT-Adverse groups (85% vs 90% females, *p* = 0.367) but patients in the latter group tended to be older (70 ± 13 vs 75 ± 9; *p* = 0.064) (Table 1).

### Persistence

**Fig. 2:**
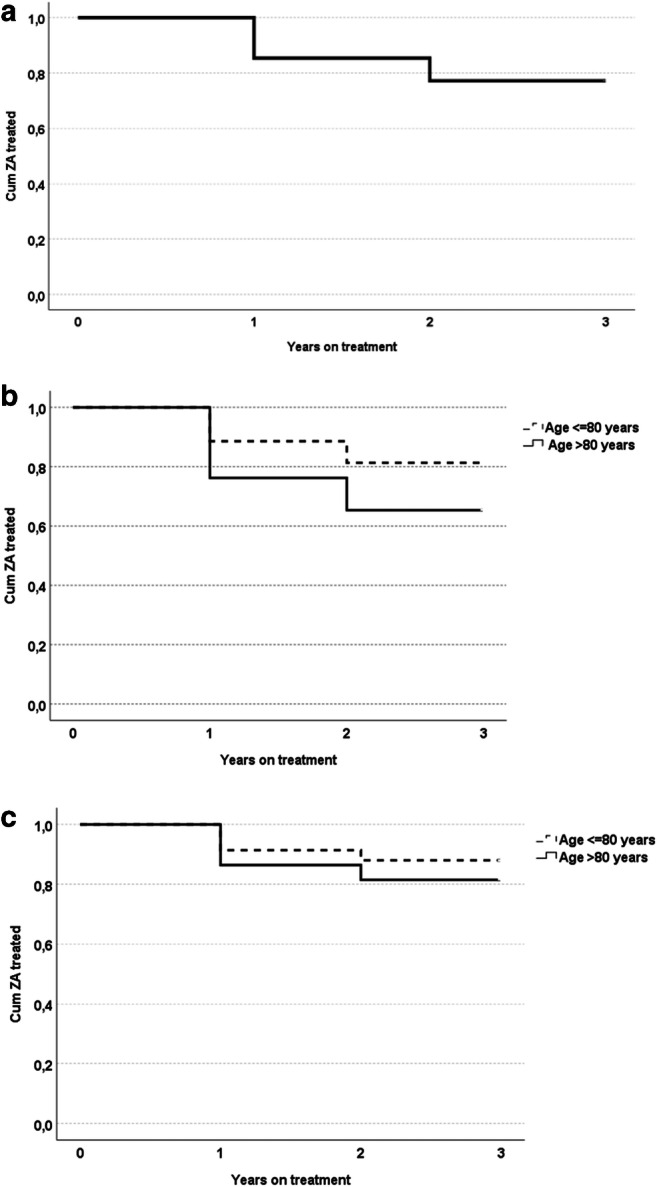
Persistence with ZA treatment. **a** All patients. **b** Patients separated into 2 age groups. **c** Patients separated into 2 age groups, and including only patients with AEs as the reason for ETT

Overall persistence rates were 85% at 2 years (i.e., with a second ZA infusion given) and 77% at 3 years (i.e., with a third ZA infusion) (Fig. 2a). With a shorter permissive time of 60 days instead of 90 days, 2- and 3-year persistence was 85% and 75%, respectively, and with a longer permissive time of 150 days, corresponding figures were 86% and 79%, respectively.

When patients were analyzed as 2 separate age categories (≤ 80 and > 80 years), persistence was lower in patients > 80 years compared to those ≤ 80 years (3-year persistence: 65% vs 81%; *p* = 0.002) (Fig. 2b). However, when the analysis was limited to the ETT-Adverse group (i.e., excluding patients with ETT because of medical reasons, aging, and other reasons), the difference between the 2 age groups in terms of 3-year persistence was smaller (82% vs 88%, *p* = 0.130) (Fig. 2c).

## Discussion

In the present study, we show that long-term adherence and persistence to ZA is high (81% and 77%, respectively) in a real-world setting organized with a tax-financed system with yearly follow-up by sending an appointment for treatment. AEs (mainly APR) were the most common reason for ETT (8%), followed by medical reasons (5%), and biologically aging (3%). ETT-Adverse was equally common in males and females but tended to be more common with increasing age.

Several studies have found low compliance with ZA; the 2-year persistence rate (i.e., with a second ZA infusion) was shown to be 25–41%, with a permissive gap of 60–90 days [8–10]. Other investigators have reported a higher rate (75% after 2 years, with a permissive gap of 112 days) [12], which is closer to our 2-year persistence data (85% with a 60- or 90-day permissive gap). There is little information on longer persistence with ZA treatment. An 80% discontinuation rate after 2 years has been observed [9]—that is, only 20% of patients received the third ZA infusion. Higher figures were reported by Tremblay et al. (54% of patients receiving ≥ 3 ZA infusions) [12]. We recorded a 3-year adherence rate of 81% and persistence rates of 77% and 75% (with 90- and 60-day grace periods, respectively), which are higher than previous findings. There are several possible reasons for the difference in the persistence rates of our cohort and rates reported in earlier studies. One is the tax-financed healthcare system in Sweden, in contrast to insurance-based systems in which treatment costs could influence patient compliance. Furthermore, logistics can vary across systems; a 3-year treatment was pre-planned for our patients, who were included on clinics’ waiting list. Because of a reminder function in this system, patients do not need to actively remember their appointment time or arrange a visit for their next ZA infusion. Furthermore, the clinic provides the ZA; thus, patients do not need to visit a pharmacy beforehand. The healthcare system in Sweden also assists with travel arrangements for patients with disabilities, thereby increasing the accessibility of the treatment program. At the visit for ZA infusion, patients are in the care of experienced osteoporosis nurses who may encourage adherence through informal education and by offering a feedback service where patients can phone with questions regarding their ZA infusion or any side effects that they experience.

In a previous study conducted in Sweden, oral bisphosphonate treatment had a low persistence rate, with just 25% of patients continuing for 3 years [7]. Thus, persistence rate varies significantly even within the same tax-financed system. In general, less frequent drug administration via the intravenous route is preferred [14] and could promote adherence.

We categorized ETT into 4 groups—namely, AE, medical reasons, biological aging, and other—so that underlying problems could be recognized and handled accordingly. Many patients who receive ZA treatment are in an age were comorbidities are common. Thus, treatment discontinuation because of biological aging and medical reasons such as reduced GFR is expected when treating this patient group. As the mean age of our patients was similar to that in other studies, our findings may also apply to those cohorts [9, 11, 12]. Patients over 80 years, sometimes referred to as the oldest old, have a high fracture burden, are often undertreated, and show a slightly different fracture risk profile compared to younger patients [6, 17]. In the present study, we show that the oldest old have a lower persistence to ZA treatment.

APR is a well-known side effect of ZA that is observed in about 40% of patients at the first infusion [16]. While it is usually mild, APR can in some cases cause more prolonged and severe symptoms. In our study, 1 in 20 ZA-treated patients (5%) terminated their treatment because of APR symptoms. These patients tended to be older than those who adhered to the treatment (mean age 75 years vs 70 years, *p* = 0.064). This finding is in disagreement with a previous report indicating that younger age is a risk factor for experiencing APR following ZA infusion [16]. However, that study examined a different outcome, i.e., the prevalence of APR post-infusion, whereas we analyzed the APRs effect on willingness for continued ZA treatment. It is possible that APRs are more severe or less tolerated in older fragile patients where APR symptoms may cause a more severe impact on daily life activity. Thus, older patients may be less willing to endure side effects compared to younger, healthier individuals, rendering a poorer adherence. Adequate hydration and NSAIDs/paracetamol are recommended for reducing APR and is routine clinical practice at our hospital. It has also been suggested that peri-infusion cortisone might prevent APR but this requires validation by additional studies.

## Conclusion

Long-term adherence to and persistence with ZA treatment was high among osteoporosis patients in Sweden, where a pre-planned therapeutic regimen is available within a tax-financed healthcare system. AEs (mainly APR) were the most common cause of ETT, occurring in about 1 of 10 patients. Further analysis of risk profiles in these patients and strategies that mitigate APR might further increase patient compliance.

## Limitations

The present retrospective study had some limitations. Firstly, reasons for ETT were obtained from medical records, which may not have included all of the reasons for patients’ decision to discontinue treatment. Secondly, the number of observations was small compared to some earlier studies. One reason for this is that, unlike most other drugs, large scale data from the national prescription registry cannot be used in the case of ZA studies in Sweden. This is because most hospitals arrange ZA infusions by having it directly delivered to the clinic, i.e., not through a pharmacy prescription on patient level. Without this prescription, patients will not be registered in the national prescription database, and thus unlike other drugs, e.g., alendronate, these patients cannot be studied using national registry data. Finally, we used local county-specific patient registers combined with clinics’ booking systems to search for patients who received ZA treatment. As stated above, these patients included most osteoporosis patients on ZA in the catchment area while excluding those who were followed at other specialized clinics (e.g., rheumatology patients). A strength of our study is that unlike in larger-scale studies, all data were confirmed by referring to case records, which included the reasons for ETT.
